# Targeted Delivery of siRNA with pH-Responsive Hybrid Gold Nanostars for Cancer Treatment

**DOI:** 10.3390/ijms18102029

**Published:** 2017-09-22

**Authors:** Hongyan Zhu, Wanwan Liu, Ziting Cheng, Ke Yao, Yu Yang, Bohui Xu, Gaoxing Su

**Affiliations:** School of Pharmacy, Nantong University, Nantong 226001, China; llw_ntu@163.com (W.L.); ztchen001@126.com (Z.C.); 18860979081@163.com (K.Y.); jackson_y233@126.com (Y.Y.); xuzi_2001@ntu.edu.cn (B.X.)

**Keywords:** gold nanostars, targeted delivery, siRNA, cancer treatment, cyclooxygenase-2, gene silence

## Abstract

In this work, we report the engineering of gold nanostars (GNS) to deliver small interfering RNA (siRNA) into HepG2 cells. The ligand DG-PEG-Lipoic acid (LA)-Lys-9R (hydrazone) was designed to functionalize GNS, and create the nanoparticles named as 9R/DG-GNS (hydrazone). In the ligand, 2-deoxyglucose (DG) is the targeting molecule, polyethylene glycol (PEG) helps to improve the dispersity and biocompatibility, 9-poly-d-arginine (9R) is employed to provide a positive surface charge and adsorb negative siRNA, and hydrazone bonds are pH-responsive and can avoid receptor-mediated endosomal recycling. Compared to GNS alone, 9R/DG-GNS (hydrazone) showed superior transfection efficiency. The expressions of cyclooxygenase-2 (COX-2) in HepG2 and SGC7901 cells were significantly suppressed by siRNA/9R/DG-GNS (hydrazone) complex. Notably, 9R/DG-GNS (hydrazone) possessed low cytotoxicity even at high concentrations in both normal cells and tumor cells. The combination treatment of siRNA/9R/DG-GNS (hydrazone) complex inhibited the cell growth rate by more than 75%. These results verified that the pH-responsive GNS complex is a promising siRNA delivery system for cancer therapy, and it is anticipated that near-infrared absorbing GNS with good photothermal conversion efficiency can be potentially used for photothermal therapy of tumors.

## 1. Introduction

Cyclooxygenase-2 (COX-2) is a rate-limiting enzyme which catalyzes the conversion of arachidonic acid to prostaglandin [1]. A large number of studies have shown that the overexpression of COX-2 is one of the signs of early tumorigenesis [2,3]. Moreover, COX-2 is also an important factor in promoting the transformation of malignant tumor cells and it is also expressed in vascular endothelial and adjacent normal tissues [1,4]. All of these findings suggest that COX-2 is an important target for tumor treatment [5,6,7]. Therefore, the down-regulation of COX-2 expression is an important research topic [5,8]. In consideration of the side effects of non-selective COX-2 steroidal drugs, which are current clinical selections [9,10], it will be of great significance to investigate the COX-2 siRNA (siCOX-2) molecule that can selectively inhibit COX-2 expression by the gene silencing effect to suppress tumor growth.

Small interfering RNA (siRNA) is used for gene suppression by mediating RNA interference (RNAi) and it is a powerful treatment for cancer [11,12,13]. However, there are several issues that limit the use of siRNA in systemic therapy [11], such as aggregation with serum proteins, degradation by endogenous nucleases, and uptake by phagocytes. In order to solve these problems, effective nanocarriers for siRNA delivery are of a great deal of interest. Recently, various gold nanomaterials have been engineered for siRNA delivery [14], such as spherical gold nanoparticles [15,16,17], gold nanorods [18,19,20,21], gold nanostars [22,23], and so on. Gold nanostars (GNS) are a new type of nanodrug carrier with the following advantages [24,25,26,27,28]: Firstly, GNS can convert the absorbed light into heat when the wavelength of light irradiation matches with the tunable surface plasmon resonance (SPR) of GNS. Secondly, the heat production capacity can be enhanced because various parts of the GNS can participate in heat production. Thirdly, suitable functional ligands can be easily combined with GNS. For example, the surface of GNS can be modified with targeting ligands to provide GNS the ability to target tumor cells. Moreover, GNS can infiltrate tumor vasculature and accumulate there because of its small size, which is conducive for increasing its penetrability into the tumor tissue [29].

2-Amino-2-deoxy-d-glucose (DG) is a kind of glucose analogue which is capable of selective combination with glucose transporter 1 (GLUT1). GLUT1 is a vital prognostic indicator of tumorigenesis [30,31,32]. When GLUT1 is present on cell membranes, it can recognize and transport DG into cells, thus DG has the effect of targeting tumor cells [33,34,35]. DG also has some effects on tumor growth; it can activate cell autophagy, and also inhibit tumors by activating the endoplasmic reticulum stress (ERS) response. Some study results have shown that DG can lead to tumor cell death by intervening in the glycolytic pathway [36]. In addition, combination of DG and antitumor drugs can efficiently improve the chemotherapy effect [37].

Based on the above considerations, we have designed and developed a pH-responsive siRNA delivery system based on GNS to target the human hepatocellular carcinoma cells (HepG2). In this system, GNS was modified with DG-polyethylene-glycol (PEG) attached to the molecules of lipoic acid (LA), lysine (Lys), and 9-poly-d-arginine (9R) by a hydrazone bond (DG-PEG-LA-Lys-9R (hydrazone)). The hydrazone bond works as a pH-responsive linkage to reveal the functional group after its cleavage in the cytoplasm and has the advantage of avoiding receptor-mediated endosomal recycling. 9R is a kind of gene carrier that delivers siCOX-2 into the cytoplasm. This carrier system could deliver siCOX-2 into the cytoplasm of HepG2 cells irreversibly to inhibit COX-2 expression and its downstream pathway so as to control the growth and metastasis of tumor cells. Moreover, in addition to efficiently targeting tumor cells, DG also plays an enhanced role in inhibiting HepG2 cell growth combined with siCOX-2. This 9R/DG-GNS system was fully characterized and then evaluated in in vitro assays as a suitable delivery system for siCOX-2.

## 2. Results and Discussion

### 2.1. Characterization of siRNA/Gold Nanostars (GNS) Complex

#### 2.1.1. The Influence of Reaction Conditions on the Growth of GNS

Based on the seed-mediated method [27,38,39], we used different synthesis methods to prepare GNS varieties with good morphology and an ultraviolet-visible-near infrared (UV-VIS-NIR) absorption peak around 808 nm for siRNA delivery and further photothermal therapy.

First, the synthesis temperature was increased (25, 40, and 60 °C) while keeping the other conditions fixed (50 μL of AA (100 mM), 100 μL of AgNO_3_ (3 mM), and 30 s reaction time). The transmission electron microscopy (TEM) images of the 25 °C group of GNS samples showed the best morphology of homogeneous stars with long, thin branches. Compared to the 25 °C group, the 40 °C group showed shorter branches, while the 60 °C group displayed a spherical shape of aggregation (Figure S1a). UV-VIS-NIR absorption peaks of the GNS samples grown at 25, 40, and 60 °C were 795, 625, and 672 nm, respectively, which indicated significant blue-shifts at higher temperatures (Figure S1b). Therefore, room temperature was chosen as the optimal synthesis temperature for GNS.

With different added amounts of AA (50, 150, and 200 μL), the morphology of GNS samples in the TEM images did not display much difference, as shown in Figure S2a. In addition, the UV-VIS-NIR absorption peaks of the 50, 150, and 200 μL groups were 792, 734, and 718 nm, respectively (Figure S2b), indicating blue-shifts with increasing AA amounts. Considering the above reasons, we chose 50 μL of AA (100 mM) to synthesize GNS.

Next, we studied the influence of different amounts (100, 200, 400, and 500 μL) of Ag^+^ (3 mM AgNO_3_) on GNS synthesis, while keeping the other conditions the same (synthesis temperature: 25 °C, AA (100 mM): 50 μL, and reaction time: 30 s). When the volume of AgNO_3_ solution (3 mM) was less than 400 μL, morphology of the GNS samples appeared almost the same in the TEM images (Figure 4a). In addition, the 500 μL group showed spherical shape of aggregation (Figure S3a). The UV-VIS-NIR peaks for GNS samples with 100, 200, 400, and 500 μL usage amounts of 3 mM AgNO_3_ were 802, 709, 751, and 690 nm, respectively (Figure S3b). The UV absorption peak of the 100 μL group is closest to 808 nm. Thus, 100 μL of 3 mM AgNO_3_ was chosen for GNS synthesis.

Finally, based on the optimum conditions above (25 °C, 50 μL of AA (100 mM), and 100 μL of AgNO_3_ (3 mM)), we observed the morphology and UV-VIS-NIR absorption of GNS at different time points during the reaction process. From the TEM images of these groups (Figure S4a), we could observe the growth process as the particles grew from seeds into GNS. The 30 s group showed the best morphology of homogeneous star with long thin branches. When the reaction time was too long (t > 6 min), the branches of GNS disappeared and uniformity was poorer. The UV-VIS-NIR absorption peaks of GNS at 0, 30 s, 2 min, 4 min, 6 min, 1 h, and 12 h were 674, 792, 731, 742, 749, 760, and 578 nm, respectively (Figure S4b). Finally, we chose 30 s as the optimum reaction time because of its largest UV-VIS-NIR absorption peak and good morphology. With all these variables carefully controlled, a successful and repeatable process for GNS synthesis was developed.

#### 2.1.2. Size Distribution, Zeta Potential, Morphology and Optical Characterization of the GNS Complex

**Figure 1:**
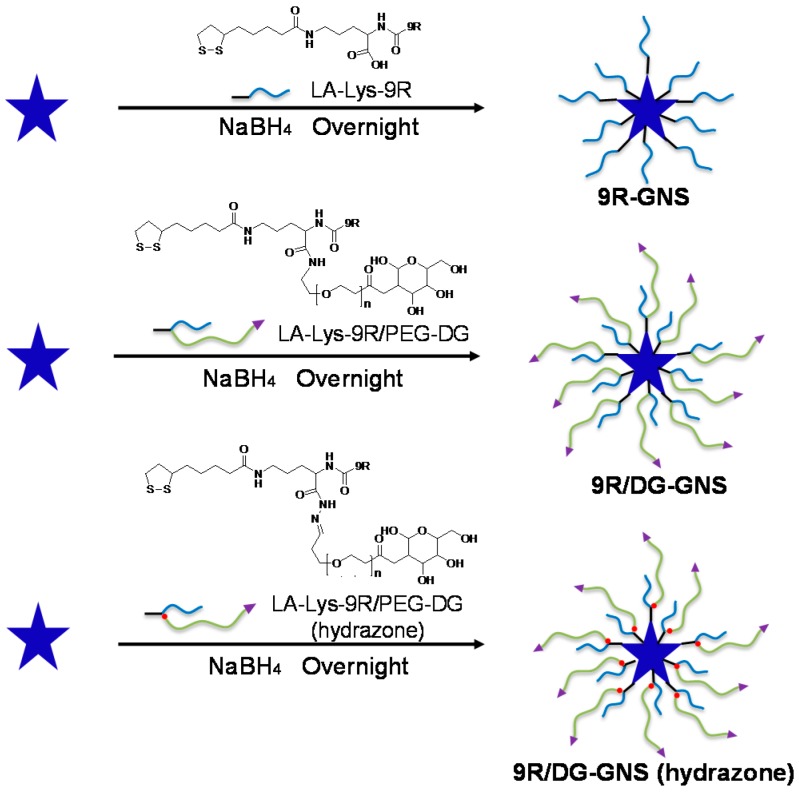
Synthesis of 9R-GNS, 9R/DG-GNS and 9R/DG-GNS (hydrazone) using ligands LA-Lys-9R, LA-Lys-9R/PEG-DG, and LA-Lys-9R/PEG-DG (hydrazone), respectively. The starting materials (the left blue star) is bared GNS, which were synthesized by seed-mediated growth method. GNS, gold nanostars; DG, 2-deoxyglucose; 9R, 9-poly-d-arginine; PEG, polyethylene glycol; LA, α-Lipoic acid.

**Figure 2:**
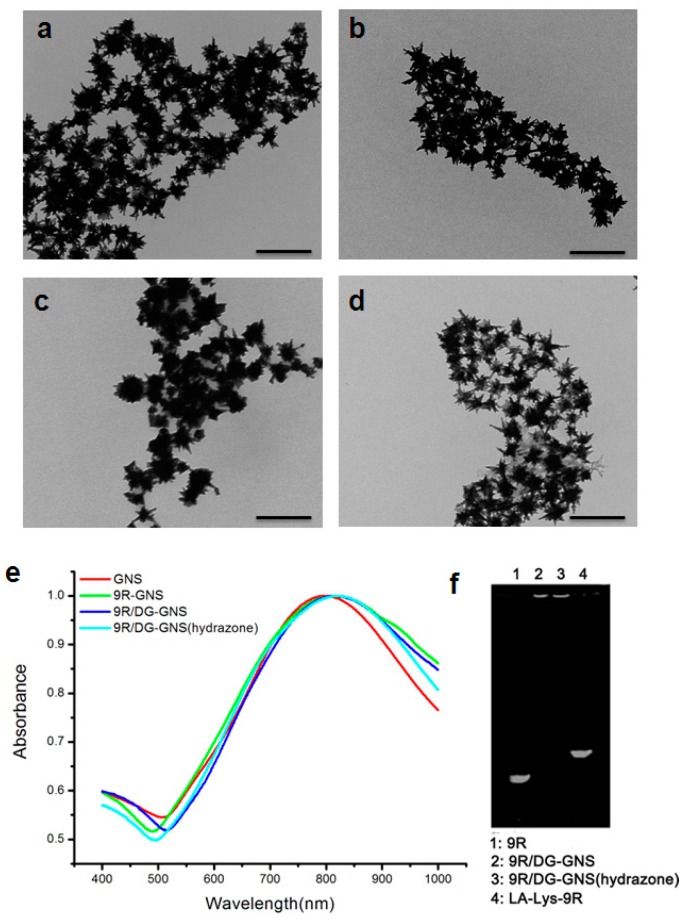
TEM images of GNS: (**a**), 9R-GNS (**b**), 9R/DG-GNS (**c**) and 9R/DG-GNS (hydrazone) (**d**). Scale bar: 200 nm; (**e**) UV-VIS-NIR absorption spectra of GNS, 9R-GNS, 9R/DG-GNS, and 9R/DG-GNS (hydrazone); and (**f**) The sodium dodecyl sulfate polyacrylamide gel electropheresis (SDS-PAGE) of 9R, 9R/DG-GNS, 9R/DG-GNS (hydrazone), and LA-Lys-9R.

For the targeted delivery of siRNA, GNS were modified with the ligand DG-PEG-LA-Lys-9R (hydrazone) to form the complex, 9R/DG-GNS (hydrazone) (Figure 1). As controls, the 9R-GNS and 9R/DG-GNS complexes were also prepared using ligands LA-Lys-9R and DG-PEG-LA-Lys-9R, respectively (Figure 1). Size distribution, polydispersity index (PDI), and zeta potentials of the GNS complex are shown in Table 1. The sizes of GNS, 9R-GNS, 9R/DG-GNS, and 9R/DG-GNS (hydrazone) were 72.5 ± 3.2, 77.8 ± 9.7, 230.7 ± 8.6, and 210.5 ± 10.3 nm, respectively. The functionalization of 9R and DG on the surface of GNS accounted for the increased size of hybrid nanoparticles in aqueous solution. Moreover, the relatively low PDI of 9R/DG-GNS and 9R/DG-GNS (hydrazone) made it possible for us to utilize them for further study. The zeta potentials of GNS, 9R-GNS, 9R/DG-GNS, and 9R/DG-GNS (hydrazone) were 2.42 ± 0.22, 15.53 ± 2.04, 11.26 ± 3.21, and 12.35 ± 2.28 mV, respectively. The sizes of GNS and 9R-GNS determined by dynamic light scattering (DLS) were in accordance with the results from TEM (Figure 2a,b). On the other hand, the sizes of 9R/DG-GNS and 9R/DG-GNS (hydrazone) obtained from the TEM images were smaller than the sizes determined by DLS (Figure 2c,d). This could be due to some amount of contraction caused by reduced moisture in the GNS complex during preparation of the TEM sample, because biological materials on the surface of GNS can display swelling. Morphologies of 9R-GNS, 9R/DG-GNS, and 9R/DG-GNS (hydrazone) (Figure 2a–d) were star-shaped with long, thin branches and showed good homogeneity and dispersibility.

The UV-VIS-NIR absorption peaks of GNS, 9R-GNS, 9R/DG-GNS, and 9R/DG-GNS (hydrazone) were 802, 808, 817, and 820 nm, respectively (Figure 2e). Considering the size distribution, it can be concluded that the UV absorption peaks showed a red-shift as the particle size became larger after chemical modification.

Sodium dodecyl sulfate polyacrylamide gel electropheresis (SDS-PAGE) was employed to characterize the attachment between the GNS and ligands. After attachment, ligand LA-Lys-9R did not move any more in the SDS-PAGE (Figure 2f).

#### 2.1.3. Analysis of Binding Ability of GNS Complex and siRNA

**Figure 3:**
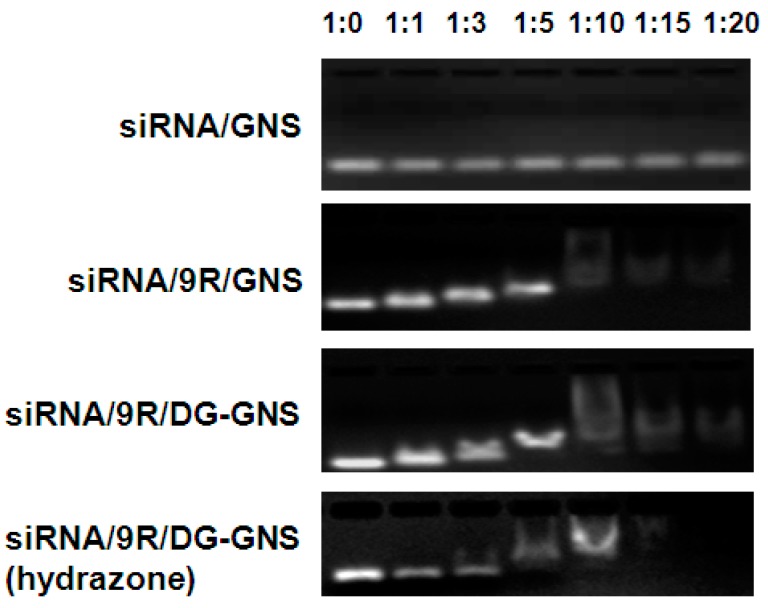
The agarose gel electrophoresis of GNS, 9R-GNS, 9R/DG-GNS, 9R/DG-GNS (hydrazone) and siRNA mixture with different ratios (1:0, 1:1, 1:5, 1:10, 1:15 and 1:20).

The sequences of siCOX-2 and negative control siRNA (siNC) are listed in Table 2. The binding ability of the GNS complex with siRNA was analyzed by agarose gel electrophoresis. Figure 3 shows that the movement of the compounds towards the anode was slow when the mole ratio of siRNA and GNS complex was 1:10, 1:15, or 1:20, which indicated good binding ability between siRNA and the GNS complex. This is because binding of appropriate amount of the GNS complex with weak positive charge and siRNA with negative charge resulted in potential neutralization. As a result, the movement of combined siRNA towards the anode was slowed down or prevented. This result shows that a suitable combination of the GNS complex and siRNA can help reduce the negative charge of siRNA.

### 2.2. Cytotoxicity of GNS Complex

**Figure 4:**
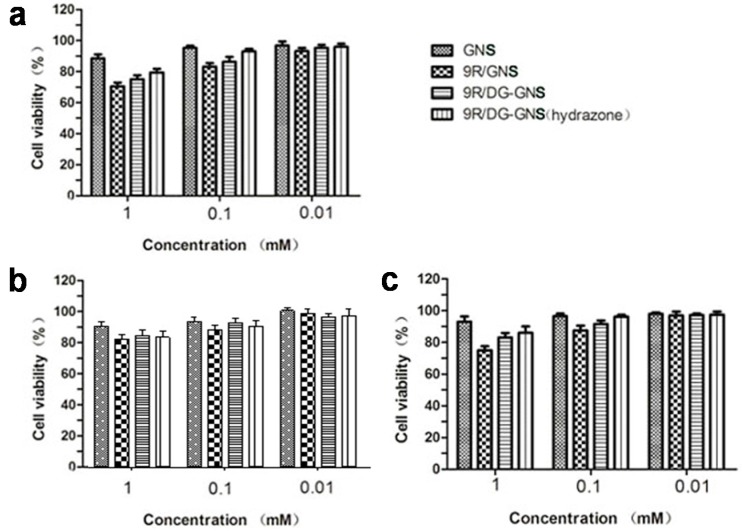
Cytotoxicity of GNS, 9R-GNS, 9R/DG-GNS, and 9R/DG-GNS (hydrazone) in HepG2 (**a**), SGC7901 (**b**), and HUVEC (**c**) cells. Data are represented as mean ± SD (*n* = 3).

In vitro cytotoxicity of the GNS complex for tumor and normal cells was studied in HepG2, SGC7901, and human umbilical vein endothelial cells (HUVEC) cells, respectively. As shown in the cell viability histogram (Figure 4), viabilities of HepG2 cells treated with GNS, 9R-GNS, 9R/DG-GNS, and 9R/DG-GNS (hydrazone) (1 mM) were 88%, 68%, 72%, and 76%, respectively, indicating relatively low cytotoxicity at the maximum concentration. When the concentration of the GNS complex was decreased to 0.01 mM, viability of HepG2 cells treated with GNS, 9R-GNS, 9R/DG-GNS, and 9R/DG-GNS (hydrazone) was more than 90% (Figure 4a). At the concentration of 1 mM, the cell viabilities of SGC7901 gastric cancer cells were higher than 80% for all treatments (Figure 4b). The viability of HUVEC cells reached more than 95% when treated with a low concentration of the GNS complex (0.01 mM). When the concentration of the GNS complex was 1 mM (maximum concentration), viabilities of HUVEC cells treated with GNS, 9R-GNS, 9R/DG-GNS, and 9R/DG-GNS (hydrazone) were 92%, 75%, 80%, and 82%, respectively (Figure 4c). In general, when the concentration of the GNS complex was low (0.01 mM), it showed no toxicity towards both normal and tumor cells. However, at high concentration (1 mM), the GNS complex showed cytotoxicity, which was likely due to the selective entry into tumor cells of the GNS complex.

### 2.3. In Vitro Cell Uptake

It is well known that the efficient delivery of antitumor drugs into tumor cells is the key point. The value of using drug carriers is the ability to change the delivery mode and in vivo distribution of drugs and deliver drugs to the target organ system, which will achieve maximum efficacy in the targeted area and reduce the side effects.

**Figure 5:**
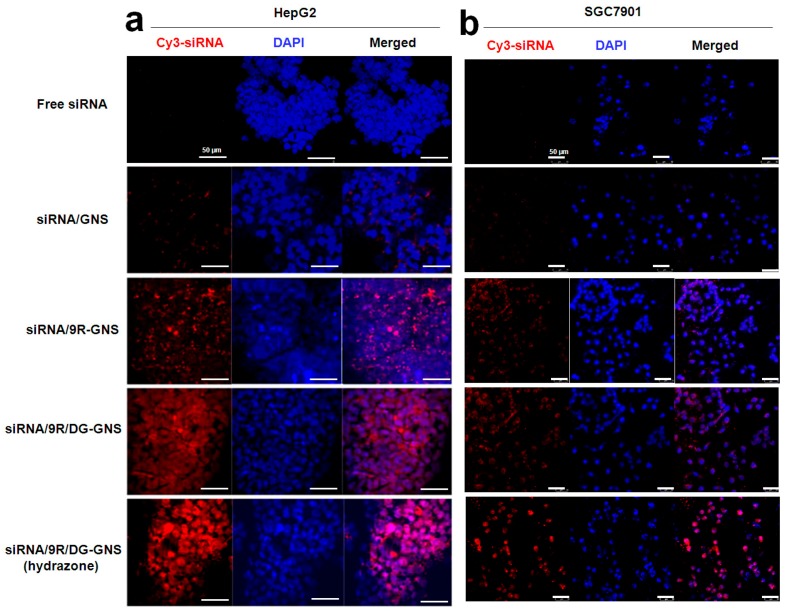
In vitro HepG2 (**a**) and SGC 7901 (**b**) cells uptake of the siRNA and GNS complex (siRNA/GNS, siRNA/9R/DG-GNS, and siRNA/9R/DG-GNS (hydrazone)) observed with laser confocal microscopy after incubation for 2 h. Scale bar: 50 μm. The red color is the fluorescence of Cy3-siRNA, and blue color is 4′,6-Diamidino-2-Phenylindole (DAPI).

Laser confocal microscopy was used to study the HepG2 and SGC7901 cell uptake of Cy3 labeled siRNA attached to GNS, 9R-GNS, 9R/DG-GNS and 9R/DG-GNS (hydrazone), respectively. As shown in Figure 5, the siRNA/GNS was actually unable to enter into HepG2 or SGC7901 cells, but siRNA/9R-GNS was partly able to enter into HepG2 or SGC7901 cells. Compared with the cellular uptake of other groups, the siRNA/9R/DG-GNS (hydrazone)-treated cells showed greater fluorescence intensity, indicating the highest cell uptake in both HepG2 and SGC7901 cells.

The negative charge of the cytomembrane prevents free siRNA from entering into the intracellular region because of the negative charge of siRNA, whereas the GNS complex carrier with positive charge can offset part of the negative charge of siRNA. Thus, the intracellular uptake of siRNA loaded in the GNS complex is increased. 9R/DG-GNS (hydrazone) also showed uptake by tumor cells due to the targeting effect of DG. Positive 9R can bind the negative siRNA, used as a gene carrier [40,41], and it has been shown to have superior transmembrane transport ability [42,43]. In addition, the hydrazone bond was destroyed in the low endosomal pH microenvironment of tumor cells. Thus, DG-PEG was released from siRNA/9R/DG-GNS (hydrazone) at the endosome and returned to the extracellular space through endocytosis. However, the 9R-GNS with siRNA remained in the cell irreversibly. Thus, the 9R/DG-GNS (hydrazone) group showed the greatest fluorescence intensity among all the groups due to the targeting effect of DG, as well as the pH-responsive hydrazone bond.

### 2.4. Gene Silencing Efficiency of siRNA/GNS Complex

Blocking or inhibiting expression of the COX-2 protein can control the growth and metastasis of tumor cells and enhance the effect of radiation and chemotherapy [6]. Therefore, the delivery of siRNA bound to a carrier with good biocompatibility and targeting ability for gene silencing has become a focus of biomedical research in recent years [44,45,46].

**Figure 6:**
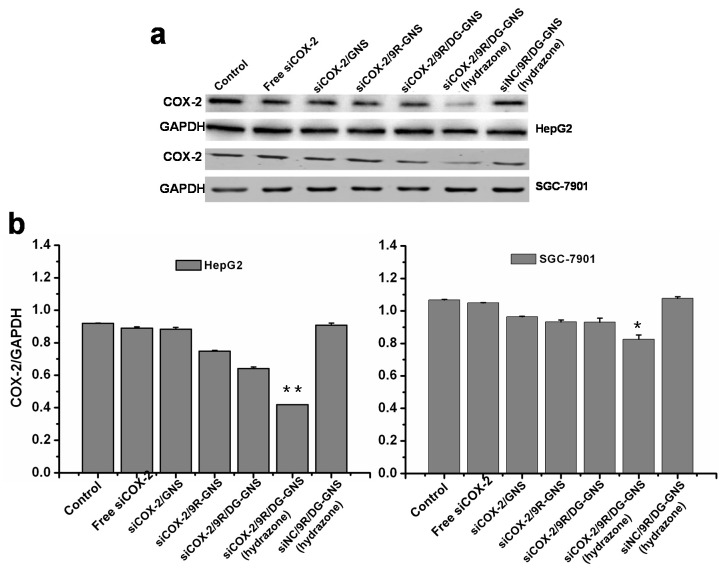
(**a**) COX-2 expressions in HepG2 and SGC7901 cells were observed with Western blot after incubation with free siCOX-2 (lane 2), siCOX-2/GNS (lane 3), siCOX-2/9R-GNS (lane 4), siCOX-2/9R/DG-GNS (lane 5), siCOX-2/9R/DG-GNS (hydrazone) (lane 6), and siNC/9R/DG-GNS (hydrazone) (lane 7) (50 nM). Lane 1 is the COX-2 expression in cells without treatment, and is marked as control. (**b**) Protein bands are quantified with the ratio of grey level of COX-2 to GAPDH. * *p* < 0.05, ** *p* < 0.01 vs. other groups. GAPDH, glyceraldehyde-3-phosphate dehydrogenase.

Gene silencing efficiencies of siRNA-GNS complex in HepG2 and SGC7901 cells were tested by Western blot experiment. As shown in Figure 6, compared to the free siCOX-2 or siNC/GNS complex groups, the down-regulation effect of COX-2 protein expression was more significant in the siCOX-2/GNS complex groups. Additionally, the siCOX-2/9R/DG-GNS (hydrazone)-treated HepG2 cells showed a greater extent of down-regulated COX-2 protein expression than other siCOX-2/GNS complex groups. DG-based complex has shown excellent potential for gene target therapy. In addition, the 9R derivative discussed earlier has high efficiency in increasing the cell uptake of siRNA. These advantages, together with the pH-responsive hydrazine, allow this nanocarrier system (siCOX-2/9R/DG-GNS (hydrazone)) to escape from endocytic recycling. siCOX-2/9R/DG-GNS (hydrazone) showed the best gene silencing efficiency which was in accordance with the best target uptake effect shown by the siCOX-2/9R/DG-GNS (hydrazone) group. Thereby, the transfection ability was improved, which led to the best gene silencing effect of siRNA loaded 9R/DG-GNS (hydrazone). The gene silencing effect in HepG2 cells was higher than that in SGC7901 cells, which was attributed from the higher density of GLUT1 on the membrane of HepG2 cells [47].

### 2.5. Effects of siRNA/GNS Complex on Cancer Cell Growth

**Figure 7:**
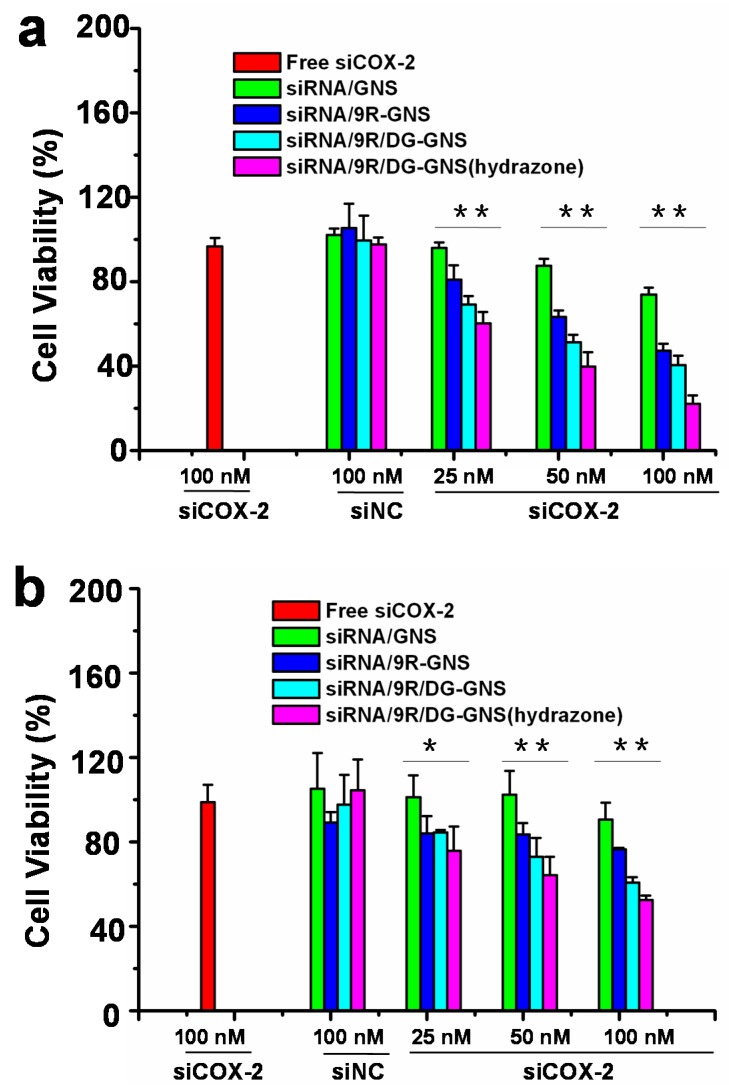
Cell viabilities of HepG2 (**a**) and SGC7901 (**b**) cells after transfected with 100 nM of siCOX-2, 25, 50 and 100 nM of siCOX-2 loaded GNS, 9R-GNS, 9R/DG-GNS, 9R/DG-GNS (hydrazone), or 100 nM of siNC loaded 9R/DG-GNS (hydrazone). Data represent mean ± SD (*n* = 4). * *p* < 0.05, ** *p* < 0.01.

Effect of GNS complex on tumor cell growth was studied by CCK-8 method, and the results are shown in Figure 7. The groups of siCOX-2/GNS, siCOX-2/9R-GNS, siCOX-2/9R/DG-GNS, and siCOX-2/9R/DG-GNS (hydrazone) complexes showed greater inhibition effect on tumor cell growth than the groups of free siCOX-2 and siNC/9R/DG-GNS (hydrazone). With increased concentration of the GNS complex (25 nM, 50 nM, 100 nM), the inhibition of tumor cell growth in all groups showed a tendency of dose-dependent increase (Figure 7). When the maximum concentration of GNS complex was 100 nM, cell viabilities of HepG2 cells were only 37%, 36%, 33%, and 24% for the groups of siCOX-2/GNS, siCOX-2/9R-GNS, siCOX-2/9R/DG-GNS, and siCOX-2/9R/DG-GNS (hydrazone), respectively. Among these, the siCOX-2/9R/DG-GNS (hydrazone) group showed the best inhibition effect on tumor cell growth in both HepG2 and SGC7901 cells. The possible reasons could be as follows: the siCOX-2/siCOX-2/9R/DG-GNS (hydrazone) has a better ability to target tumor cells efficiently compared to siCOX-2 alone; and siCOX-2 can enter into intracellular space irreversibly after the pH-responsive hydrazone bond of siCOX-2/9R/DG-GNS (hydrazone) was destroyed. The results further verified and illustrated the antitumor activity of siRNA-GNS complex. Thereby, this pH-responsive gene nanocarrier system is useful for siRNA delivery in order to achieve gene therapy. Moreover, this nanocarrier system can potentially be applied to the combination of gene therapy and photothermal therapy by making use of the photothermal conversion effect of GNS.

## 3. Materials and Methods

### 3.1. Materials

Hydrogen tetrachloroaurate tetrahydrate (HAuCl_4_∙4H_2_O), silver nitrate (AgNO_3_), sodium borohydride (NaBH_4_), ascorbic acid (AA), and α-lipoic acid (LA) were purchased from Sinopharm Chemical Reagent Inc. (Shanghai, China). Dicyclohexylcarbodiimide (DCC), *N*-hydroxysuccinimide (NHS), Boc-l-Lysine (Lys) and 2-amino-2-deoxy-d-glucose (DG) were purchased from Aladdin Reagent Inc. (Shanghai, China). Polyethylene glycol (PEG, molecular weight: 5000) was purchased from Yanyi Biology and Technology Inc. (Shanghai, China). 9-d-Arginine (9R) was purchased from GL Biochemical Ltd. (Shanghai, China). Dulbecco's modified eagle’s medium (DMEM)/F12 culture medium, RPMI-1640 culture medium and fetal bovine serum were purchased from Gibco (San Diego, CA, USA). Trypsin (0.5%), SDS-PAGE kit, RIPA lysis buffer, and primary antibody dilution buffer were purchased from Beyotime Institute of Biotechnology (Nantong, China). COX-2 antibody was purchased from Cell Signaling Technology (Danvers, MA, USA). GAPDH antibody was purchased from Sigma-Aldrich (St. Louis, MO, USA). Goat anti-rabbit IgG-phycoerythrin (IgG-PE) second antibody was purchased from Santa Cruz Biotech (Santa Cruz, CA, USA). Cox-2 siRNA (sense strand, 5′-AACUGCUCAACACCGGAA-3′, antisense strand 5′-AUUCCGGUGUUGAGCAGU-3′) and NC siRNA (sense strand, 5′-UUCUCCGAACGUGUCACGU-3′, antisense strand 5′-ACGUGACACGUUCGGAGA-3′) were obtained from Biomics Biotechnologies (Nantong, China).

### 3.2. Methods

#### 3.2.1. Synthesis of Ligands LA-Lys-9R, LA-Lys-9R/PEG-DG, and LA-Lys-9R/PEG-DG (Hydrazone)

The synthesis and characterization of ligands LA-Lys-9R, LA-Lys-9R/PEG-DG, and LA-Lys-9R/PEG-DG (hydrazone) have been reported in our previous work [47].

#### 3.2.2. Preparation and Characterization of GNS

##### Synthesis of Gold Seeds

Aqueous solution of 1% sodium citrate (15 mL) was added to boiling HAuCl_4_ solution (100 mL, 1 mM) under vigorous stirring and allowed to react for 15 min. After cooling down, the solution was filtered through a membrane with 0.22 μm pore size. The obtained solution was used as gold seeds in the next step and could be kept under 4 °C for long-term storage.

##### Synthesis of GNS

GNS were synthesized by seeded growth method [38]. Typically, 100 μL of the seeds solution was added to the HAuCl_4_ solution (10 mL, 0.25 mM). Then, 10 μL 1 M HCl was added to the mixture. With vigorous stirring at room temperature, AA (100 mM) and AgNO_3_ (3 mM) were added to the mixture at the same time. The solution color changed rapidly from light red to blue, indicating the successful formation of GNS. To investigate the effects of reaction conditions on the growth of GNS, three different reaction temperatures (25, 40, 60 °C), three different volumes of 100 mM AA (50, 150, 200 μL), four different volumes of 3 mM AgNO_3_ (100, 200, 400, 500 μL), and different reaction times (0, 30 s; 2 min, 4 min, 6 min; 1 h, 12 h) were employed to conduct the synthesis.

##### Characterization of GNS

TEM (JEM-1011, operating at 100 kV, JEOL, Tokyo, Japan) was used to observe the morphology of GNS synthesized under different reaction conditions. UV-VIS-NIR spectra of GNS synthesized under different reaction conditions were obtained by 754PC spectrometer (Jinghua Instruments, Shanghai, China).

#### 3.2.3. Preparation and Characterization of Hybrid GNS Complex

The selected ligand (LA-Lys-9R, LA-Lys-9R/PEG-DG, or LA-Lys-9R/PEG-DG (hydrazone)) (0.05 mmol) was dissolved in ethanol and water (volume ratio 1:1). Next, NaBH_4_ (0.75 mmol) was added as the reductant and stirred for 4 h. Then, GNS solution (10 mL) was added to the mixture dropwise at room temperature. After stirring overnight, the obtained solution was centrifuged at 10,000 rpm for 30 min. The pellet was washed with ethanol and water several times and dispersed in 1 mL water to give the stock solution of the GNS complex.

The hydrodynamic diameters, PDI, and zeta potentials of GNS, 9R-GNS, 9R/DG-GNS, and 9R/DG-GNS (hydrazone) were measured using Malvern Zetasizer Nano ZS90 (Malvern Instrument Ltd., Malvern, UK). The size and morphology of hybrid GNS complex were observed with TEM (JEM-1011, operating at 100 kV). UV-VIS-NIR spectra of hybrid GNS were recorded on a 754PC spectrophotometer (Jinghua Instruments, Shanghai, China).

#### 3.2.4. Preparation and Characterization of the Mixture of siRNA and GNS Complex

siCOX-2 was mixed with GNS, 9R-GNS, 9R/DG-GNS and 9R/DG-GNS (hydrazone) separately according to the molar ratios of 1:0, 1:1, 1:5, 1:10, 1:15, and 1:20 at 4 °C for 45 min. Afterwards, 4% agarose gel electrophoresis was used to analyze the binding ability between siCOX-2 and GNS, 9R-GNS, 9R/DG-GNS, and 9R/DG-GNS (hydrazone). In detail, a solution of 4% agarose gel was heated in a microwave oven for about 1 min for dissolving, then 3 μL 10,000× GelRed^TM^ nucleic gel stain was added to the solution immediately and mixed adequately. The gel liquid was then added into the mold, and the comb was inserted. After protecting from light for 30 min, the comb was removed, agarose gel was placed into the electrophoresis tank, and 0.5× TBE electrophoretic buffer solution was added to submerge the gel. The previously-prepared samples were loaded and the voltage was kept constant at 120 V for around 20 min for the electrophoresis. Then, the agarose gel was removed and pictures of the gel were taken for analysis by a gel imaging system.

#### 3.2.5. Cell Experiments

HepG2, SGC7901 and HUVEC cells were cultured in RPMI 1640 medium with 10% fetal bovine serum (FBS), penicillin (100 U/mL), and streptomycin (100 µg/mL) in a humidified condition with 5% CO_2_ at 37 °C.

##### 3-(4,5-Dimethylthiazol-2-yl)-2,5-diphenyltetrazolium Bromide (MTT) Assays

The HepG2, SGC7901 or HUVEC cells were seeded with the density of 5000 cells/well in a 96-well plate. After 24 h culturing, the culture medium was replaced with 150 µL serum-free medium of blank group or complete medium of control group. The other groups were replaced with solutions of GNS, 9R-GNS, 9R/DG-GNS, and 9R/DG-GNS (hydrazone) (three concentrations for every solution: 10, 100, and 1000 μM) respectively, and the cells were cultured for 48 h in an incubator (37 °C, 5% CO_2_). Thereafter, the medium was removed and 20 µL MTT solution was added to each well and cultured continuously for 4 h. Then MTT was removed and 150 μL DMSO was added to each well followed by slight shaking for 10 min. Viability of the cells was analyzed by optical density (OD) detected by a Thermo Scientific Microplate Reader (TecanInc, Männedorf, Switzerland) at 490 nm.

##### In Vitro Cellular Uptake

We seeded 50,000 HepG2 or SGC7901 cells per well in 24 well plates with cover glass and cultured the cells for 24 h in an incubator (37 °C, 5% CO_2_). Then, Cy3 labeled siRNA (50 nM) was mixed with GNS, 9R-GNS, 9R/DG-GNS, or 9R/DG-GNS (hydrazone) (molar ratio of siRNA and GNS complex is 1:20) and the mixtures were kept on ice for 45 min. The cover glass was taken out and washed with phosphate buffered saline (PBS, 0.01 M, pH 7.2) thrice for 5 min. The mixed samples were added to the wells and cultured in an incubator (37 °C, 5% CO_2_) for 2 h. Afterwards, the wells were washed with PBS (0.01 M, pH 7.2) thrice for 5 min. Next, 4′,6-Diamidino-2-Phenylindole (DAPI) was added to the wells, and after 20 min, the wells were washed with PBS (0.01 M, pH 7.2) thrice for 5 min. Then, the cover glass was dehydrated and dried with gradient alcohol, and 50% glycerinum was used for mounting; this step was conducted away from light. Thereafter, a Leica TCS SP5 confocal laser (Leica, Buffalo Grove, IL, USA) scanning microscope was used to observe the localization of the samples. The excitation wavelengths of DAPI and Cy3-labeled siRNA are 450 and 550 nm, respectively.

##### Western Blot Analysis

The Western blot experiment was conducted to verify COX-2 expression in HepG2 or SGC7901 cell line groups treated with different concentrations of free siCOX-2 and GNS complexes (siCOX-2/GNS, siCOX-2/9R-GNS, siCOX-2/9R/DG-GNS, siCOX-2/9R/DG-GNS (hydrazone), and siNC/9R/DG-GNS (hydrazone)). In detail, we extracted proteins from cancer cells with lysis buffer. The protein concentration of the supernatant was determined by BCA assay. Sodium dodecyl sulfate polyacrylamide gel electrophoresis (SDS-PAGE) was performed by loading equal amounts of proteins per lane. Gels were then transferred to poly (vinylidene difluoride) membranes (Millipore, USA) covered by ice bags at 260 mA for 90 min. Then, the membranes were blocked with 5% non-fat milk in TBST buffer (996 mL H_2_O, 6.057 g Tris, 8.5 g NaCl, 0.05% Tween 20, pH 7.5–7.6) overnight at 4 °C on the table concentrator. Next, the membranes were incubated with primary antibodies at required dilutions in 5% non-fat milk in TBST at 4 °C inside hybridization bags overnight, followed by washing thrice with TBST for a total of 30 min. After that, the secondary antibodies were incubated at required dilutions in 1% non-fat milk in TBST at room temperature away from light for 2 h. Finally, the blots were visualized with Odyssey Clx Western blot detection system (LI-COR Biosciences, Lincoln, NE, USA).

##### CCK-8 (Cell Counting Kit-8) Method to Analyze the Effect of siRNA/GNS Complex on Cancer Cells Growth

We studied the effects of siRNA/GNS complex on the viability of HepG2 cells by the CCK-8 method. Briefly, HepG2 or SGC7901 cells (5000/well) were cultured continuously in 96-well plates, then the cells were treated with free siCOX-2 (100 nM), siNC/GNS complex solution (siNC-GNS, siNC/9R-GNS, siNC/9R/DG-GNS, and siNC/9R/DG-GNS (hydrazone) (100 nM)), and siCOX-2/GNS complex solution (siCOX-2-GNS, siCOX-2/9R-GNS, siCOX-2/9R/DG-GNS, and siCOX-2/9R/DG-GNS (hydrazone) (25 nM, 50 nM, 100 nM)). In addition, one blank group was added (containing culture medium only). Five repetitions were made for every group. After being incubated in an incubator (37 °C, 5% CO_2_) for 24 h, CCK-8 (10 μL per well) was added and the cells were continuously incubated in the incubator (37 °C, 5% CO_2_) for 2 h. Afterwards, a Thermo Scientific Microplate Reader (Tecan Inc., Männedorf, Switzerland) was used to measure the absorbance values of different samples at 450 nm.

### 3.3. Statistical Analysis

Student’s *t*-test for independent means was used to assess the significance of differences between two groups; differences between more than two groups were assessed by one-way ANOVA. * *p* < 0.05 and ** *p* < 0.01 were used for statistical significance. All data were presented as mean ± SD.

## 4. Conclusions

In this work, we have synthesized GNS with good morphology and biocompatibility through a seed-mediated method. Then, a DG, 9R modified GNS complex nanocarrier system (9R/DG-GNS (hydrazone)) was constructed successfully. Superior binding between GNS complex nanocarrier system and siRNA was observed. siCOX-2/9R/DG-GNS (hydrazone) showed good uptake by tumor cells through its targeting effect. The hydrazone bond was easily cleaved in the lysosome environment with low pH so as to release DG-PEG from the carrier and return it to the extracellular space through endocytosis, while the siCOX-2/9R-GNS remained in the intracellular space irreversibly. Thus, the GNS complex nanocarrier system can be used to deliver siCOX-2 into tumor cells efficiently. The ability of siCOX-2 for gene silencing effect and inhibiting tumor cells growth in vitro were enhanced effectively. In summary, this pH-responsive GNS complex is a promising siRNA delivery system for tumor therapy, and it is further anticipated that near-infrared absorbing GNS with good photothermal conversion efficiency can be potentially used for photothermal therapy of tumor tissues.

## Figures and Tables

**Table 1:** Characterization of the hydrodyamic diameters and zeta potentials of nanoparticles. Values were expressed as mean ± SD (*n* = 3). GNS, gold nanostars; DG, 2-deoxyglucose; 9R, 9-poly-d-arginine.

Samples	Hydrodynamic Diameter (nm)	Polydispersity	Zeta Potential (mV)
GNS	72.5 ± 3.2	0.303 ± 0.024	2.42 ± 0.22
9R-GNS	77.8 ± 9.7	0.406 ± 0.020	15.53 ± 2.04
9R/DG-GNS	230.7 ± 8.6	0.251 ± 0.013	11.26 ± 3.21
9R/DG-GNS (hydrazone)	210.5 ± 10.3	0.231 ± 0.030	12.35 ± 2.28

**Table 2:** The sequences of siRNA.

Type of siRNA	Sense (5′-3′)	Antisense (5′-3′)
COX-2 siRNA	AACUGCUCAACACCGGAA	AUUCCGGUGUUGAGCAGU
NC siRNA	UUCUCCGAACGUGUCACGU	ACGUGACACGUUCGGAGA

## Supplementary Materials

Supplementary materials can be found at www.mdpi.com/1422-0067/18/10/2029/s1.

## Abbreviations

GNS	Gold nanostar
COX-2	Cyclooxygenase-2
siRNA	Small interfering RNA
siCOX-2	COX-2 siRNA
DG	2-Amino-2-deoxy-d-glucose
PEG	Polyethylene glycol
9R	9-d-arginine
LA	α-Lipoic acid
GLUT1	Glucose transporter 1
TEM	Transmission electron microscopy
AA	Ascorbic acid
DLS	Dynamic light scattering
PDI	Polydispersity index
HUVEC	Human umbilical vein endothelial cells
HepG2	Human hepatocellular liver carcinoma
MTT	3-(4,5-dimethylthiazol-2-yl)-2,5-diphenyltetrazolium bromide
CCK-8	Cell counting kit-8
Lys	Lysine
UV-VIS-NIR	Ultraviolet-visible-near infrared
siNC	Negative control siRNA
SDS-PAGE	Sodium dodecyl sulfate polyacrylamide gel electropheresis
