# Supplementary material for: Targeted Delivery of siRNA with pH-Responsive Hybrid Gold Nanostars for Cancer Treatment
Source: Int J Mol Sci. 2017 Sep 22;18(10):2029. doi: 10.3390/ijms18102029 (PMC5666711; doi:10.3390/ijms18102029)
Supplement: Supplementary file 1 [file ijms-18-02029-s001.pdf]

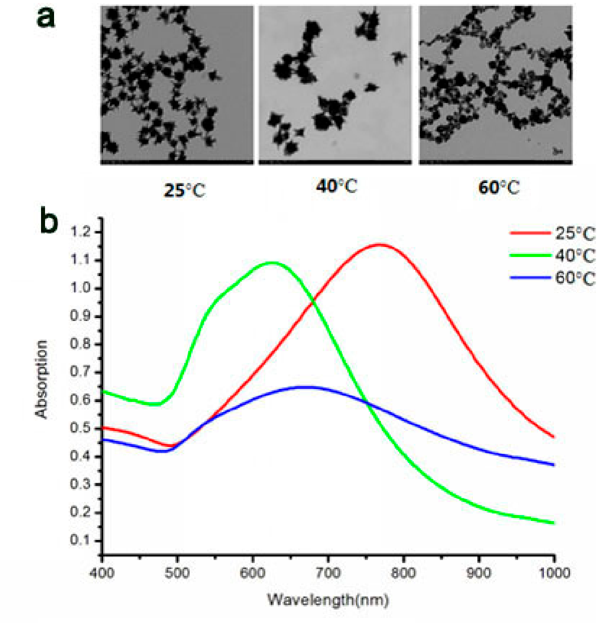

Figure S1. (**a**) TEM images and (**b**) UV absorption spectra of gold nanostars (GNS) with different reaction temperature (25 °C, 40°C, and 60 °C).

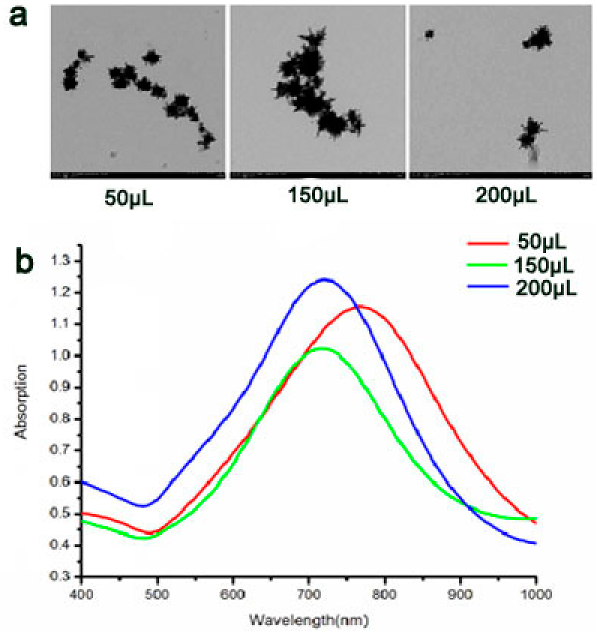

Figure S2. (**a**) TEM images and (**b**) UV absorption spectra of GNS with different amounts of reducing agent (50, 150, and 200 μL AA (0.1 M)).

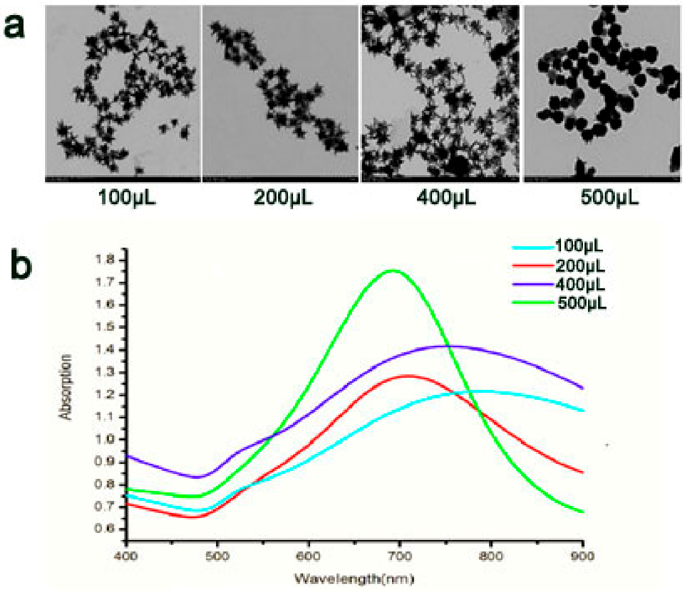

Figure S3. (**a**) TEM images and (**b**) UV absorption spectra of GNS with different amount of Ag^{+} (100, 200, 400, and 500 μL $AgNO_3$ (3 mM)).

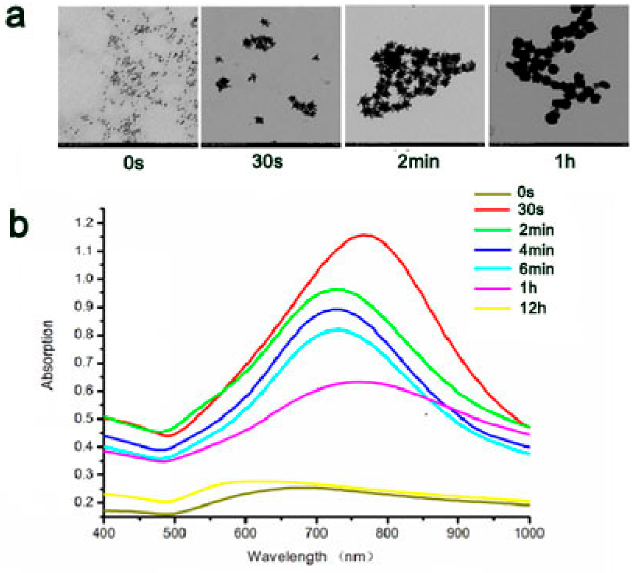

Figure S4. (**a**) TEM images and (**b**) UV absorption spectra of GNS with different reaction times (0 and 30 s; 2, 4, and 6 min; 1 h, and 12 h).
